# Development of a nomogram model for prediction of new adjacent vertebral compression fractures after vertebroplasty

**DOI:** 10.1186/s12893-023-02068-6

**Published:** 2023-07-10

**Authors:** Yadong Qian, Xiao Hu, Chen Li, Jingwei Zhao, Yanjing Zhu, Yan Yu, Ning Xie, Bin Ma, Zhili Zeng, Liming Cheng

**Affiliations:** 1 Key Laboratory of Spine and Spinal Cord Injury Repair and Regeneration of Ministry of Education, Tongji University, Shanghai, China; 2 Division of Spine, Department of Orthopaedics, Tongji Hospital, Tongji University School of Medicine, Shanghai, 200065 China

**Keywords:** Osteoporotic vertebral compression fracture, Vertebroplasty, Refracture, Risk factors, Prediction model

## Abstract

**Background:**

Vertebroplasty is the main minimally invasive operation for osteoporotic vertebral compression fracture (OVCF), which has the advantages of rapid pain relief and shorter recovery time. However, new adjacent vertebral compression fracture (AVCF) occurs frequently after vertebroplasty. The purpose of this study was to investigate the risk factors of AVCF and establish a clinical prediction model.

**Methods:**

We retrospectively collected the clinical data of patients who underwent vertebroplasty in our hospital from June 2018 to December 2019. The patients were divided into a non-refracture group (289 cases) and a refracture group (43 cases) according to the occurrence of AVCF. The independent predictive factors for postoperative new AVCF were determined by univariate analysis, least absolute shrinkage and selection operator (LASSO) logistic regression, and multivariable logistic regression analysis. A nomogram clinical prediction model was established based on relevant risk factors, and the receiver operating characteristic curve (ROC), calibration curve, and decision curve analysis (DCA) were used to evaluate the prediction effect and clinical value of the model. After internal validation, patients who underwent vertebroplasty in our hospital from January 2020 to December 2020, including a non-refracture group (156 cases) and a refracture group (21 cases), were included as the validation cohort to evaluate the prediction model again.

**Results:**

Three independent risk factors of low bone mass density (BMD), leakage of bone cement and “O” shaped distribution of bone cement were screened out by LASSO regression and logistic regression analysis. The area under the curve (AUC) of the model in the training cohort and the validation cohort was 0.848 (95%CI: 0.786–0.909) and 0.867 (95%CI: 0.796–0.939), respectively, showing good predictive ability. The calibration curves showed the correlation between prediction and actual status. The DCA showed that the prediction model was clinically useful within the whole threshold range.

**Conclusion:**

Low BMD, leakage of bone cement and “O” shaped distribution of bone cement are independent risk factors for AVCF after vertebroplasty. The nomogram prediction model has good predictive ability and clinical benefit.

## Background

In China, the prevalence of osteoporosis among people aged 40 years and older is 5.0% among men and 20.6% among women [1]. By 2050, the total number of osteoporosis patients in China is expected to reach 212 million [2]. The loss of bone mass represents a higher risk of fragility fracture [3]. Of all osteoporotic fractures, osteoporotic vertebral compression fracture (OVCF) is the most common fracture type, accounting for nearly 50% [4]. Vertebral fractures cause significant back pain and restricted movement, reducing the quality of life of elderly patients. Long-term bed rest also increases the risk of adverse events such as dropdown pneumonia, deep vein thrombosis, and bedsores, which pose a serious burden on families and society [5]. Vertebroplasty was first proposed by Galibert and applied in the treatment of vertebral hemangioma. Since then, it has been applied in the treatment of OVCF caused by osteoporosis, myeloma, and trauma. Compared with conservative therapy, vertebroplasty is a safe and effective procedure with the advantages of rapid pain relief and short recovery time [6, 7].

Although vertebroplasty provides rapid pain relief and functional recovery, some patients will present with complications including refracture, spinal cord compression, infection, nerve root injury, and embolism. The most widely investigated complication is adjacent vertebral compression fracture (AVCF) with an incidence of 6.8–37.5% [8]. Some of these patients require further treatment, causing an additional financial burden. The incidence of AVCF after vertebroplasty may be influenced both by patient characteristics and operative factors. Risk factors for AVCF have been identified, including low bone mineral density (BMD), bone cement distribution, bone cement leakage, vertebral height restoration, and number of treated vertebrae [2, 9–11]. However, the results of studies are often inconclusive or contradictory. Moreover, there are few clinical risk prediction models for AVCF after vertebroplasty which makes it difficult for clinicians to effectively manage OVCF patients. This study aims to retrospectively analyze the risk factors for new AVCF after vertebroplasty and establish a clinical prediction model, so as to guide clinical treatment.

## Materials and methods

### Study cohorts

We retrospectively collected and analyzed clinical data from patients undergoing vertebroplasty in our hospital between June 2018 and December 2020. The inclusion criteria included the following: (1) patients with low back pain and radiographic diagnosis of fresh vertebral fracture (the signal change of the lumbar fracture by lumbar magnetic resonance imaging [MRI] suggesting a hyperintense T2 signal and a hypointense T1 signal, or a whole body bone scan suggesting active bone metabolism);(2) patients who met the diagnostic criteria for osteoporosis (A or B): (A) Dual energy X-ray absorptiometry (DXA) showing T ≤-2.5 at spine/hip. (B) Sagittal L1-Hounsfield unit value ≤ 110 on computed tomography scan;(3) patients with OVCF caused by low energy injury; and (4) patients who underwent vertebroplasty. The exclusion criteria were as follows: (1) patients with OVCF caused by tumor, infection, or tuberculosis; (2) patients with OVCF caused by severe violence; (3) patients who had spinal cord compression and obvious neurological symptoms, such as numbness or muscle weakness; (4) patients who cannot tolerate surgery due to coagulation dysfunction or systemic diseases; and (5) incomplete clinical data.

**Fig. 1:**
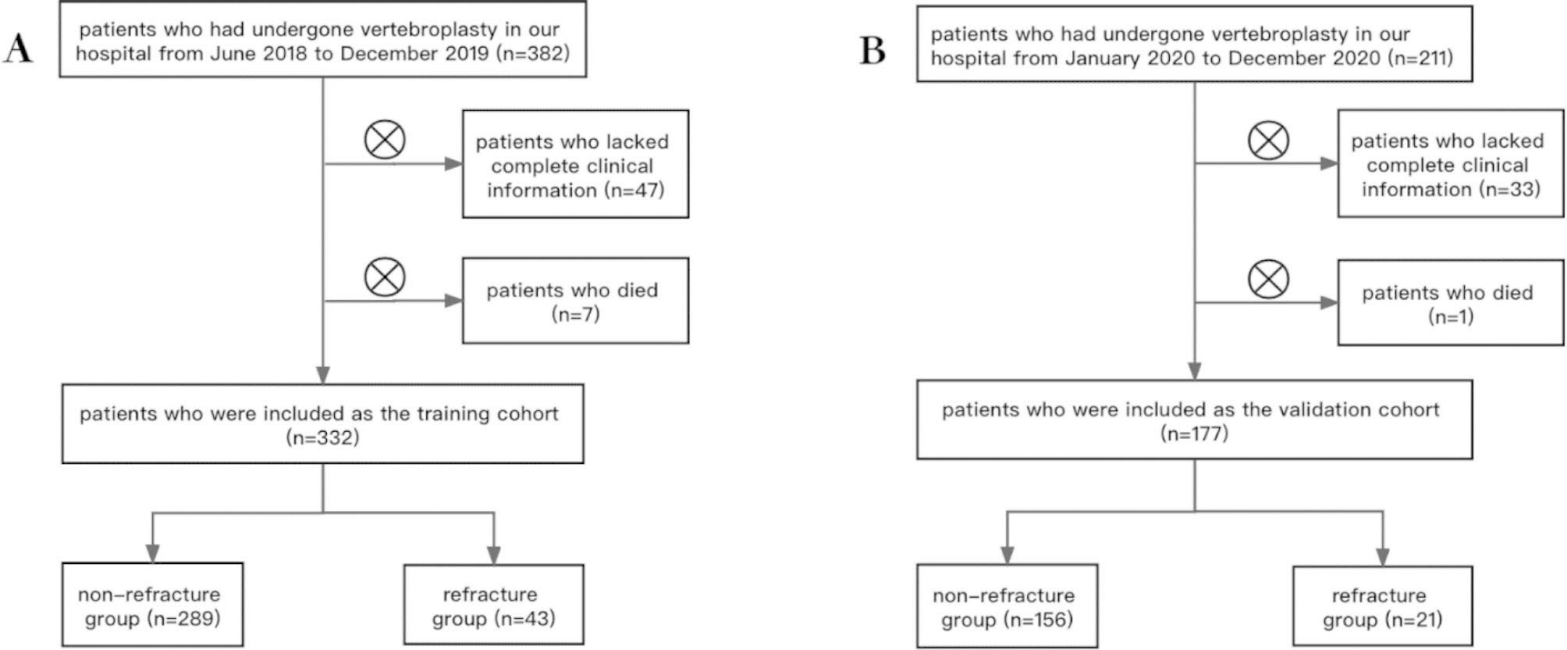
Flow diagrams show the pathway of patient inclusion and exclusion. (**A**) the training cohort, (**B**) the validation cohort

This retrospective study initially included 386 patients who had undergone vertebroplasty in our hospital from June 2018 to December 2019. We excluded 47 patients who lacked complete clinical information and 7 patients who died during the follow-up. Finally, 332 patients were included as the training cohort. These patients were divided into a non-refracture group (289 cases) and a refracture group (43 cases) according to the occurrence of AVCF during the follow-up.The median follow-up time of training cohort was 33.39(28.88 ~ 38.09)months and the median timing of AVCF was 6.03~3.00 ~ 12.20~months. Another sample of 177 patients who undergone vertebroplasty in our hospital from January 2020 to December 2020 was included as a validation cohort to evaluate the prediction model again, comprising a non-refracture group (156 cases) and a refracture group (21 cases) after excluding 34 patients with similar criteria (33 patients lacked complete clinical information and 1 patient died ). The median follow-up time of validation cohort was 17.77~15.04 ~ 20.50~months and the median timing of AVCF was 2.07~0.87 ~ 8.89~months(Fig. 1). Previous studies reported that AVCF occurred mainly within 1 year or even half a year after surgery, so we set the minimum follow-up time at 1 year. Most of the patients in our study had a refracture within six months or even two weeks, so we define the AVCF as a binary outcome.This study was approved by the Ethics Committee of the Shanghai Tongji Hospital.

### Observation factors

**Fig. 2:**
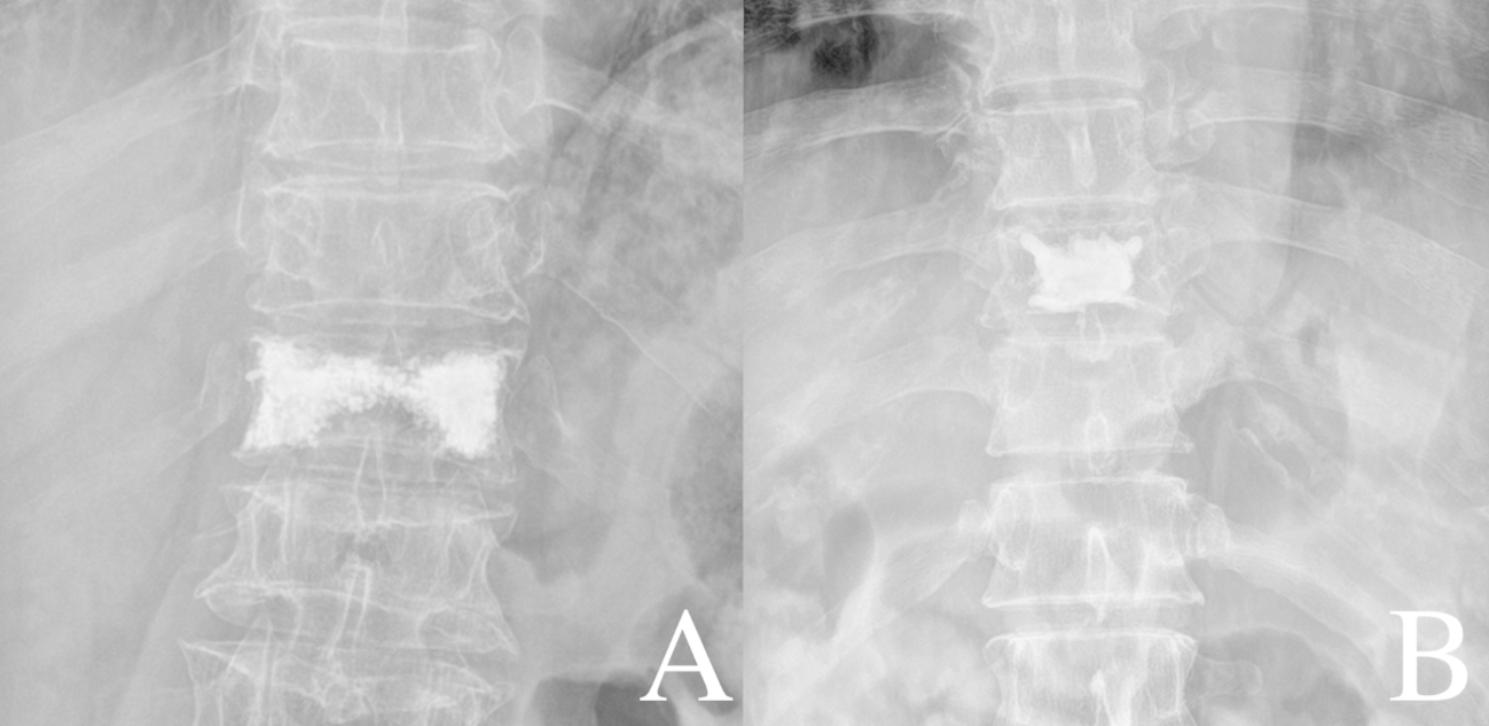
Two shapes of bone cement. **(A)** “H” shaped bone cement. **(B)** “O” shaped bone cement

**Fig. 3:**
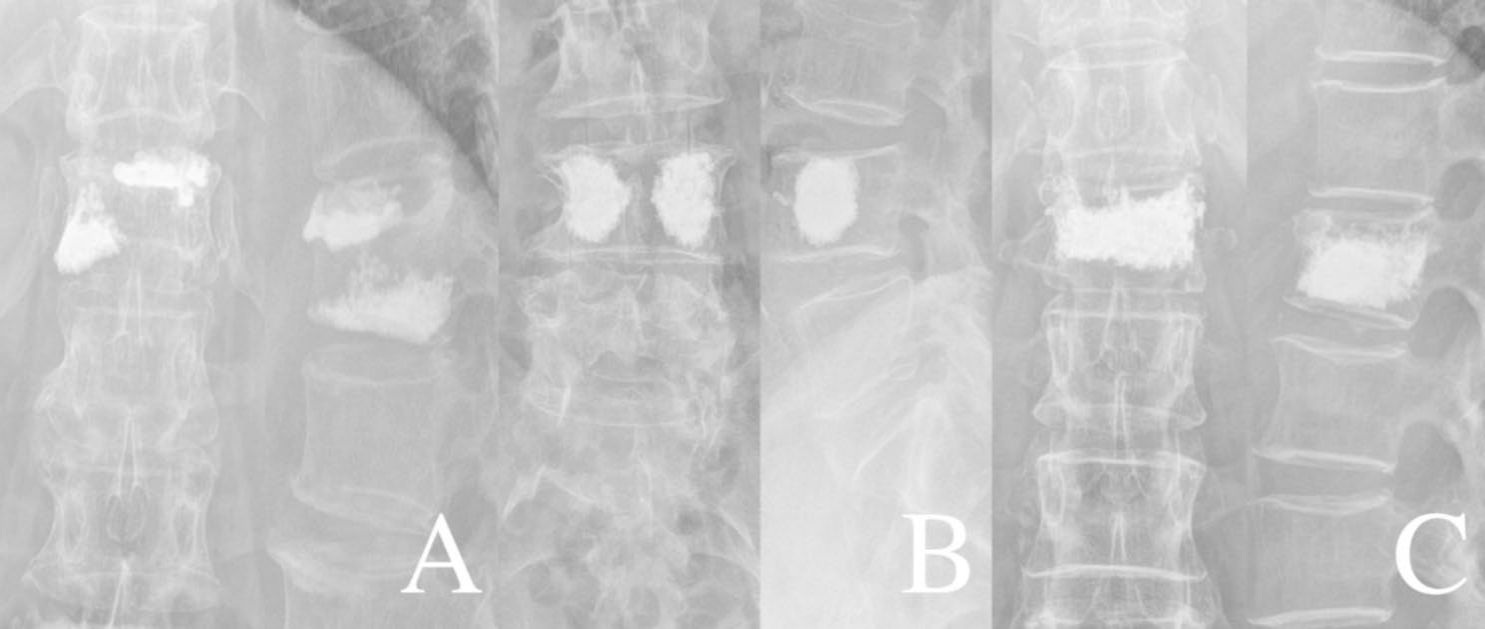
Dispersion distribution grade of bone cement. **(A)** The bone cement projection of the anteroposterior and lateral X-ray after surgery is ≤ ½, which is grade (1) **(B)** The bone cement projection of the anteroposterior was ≥ ½ and the lateral X-ray after surgery is ≤ ½, which is grade (2) **(C)** The bone cement projection of both the anteroposterior and lateral X-ray after surgery were ≥ ½, which is grade 3

**Fig. 4:**
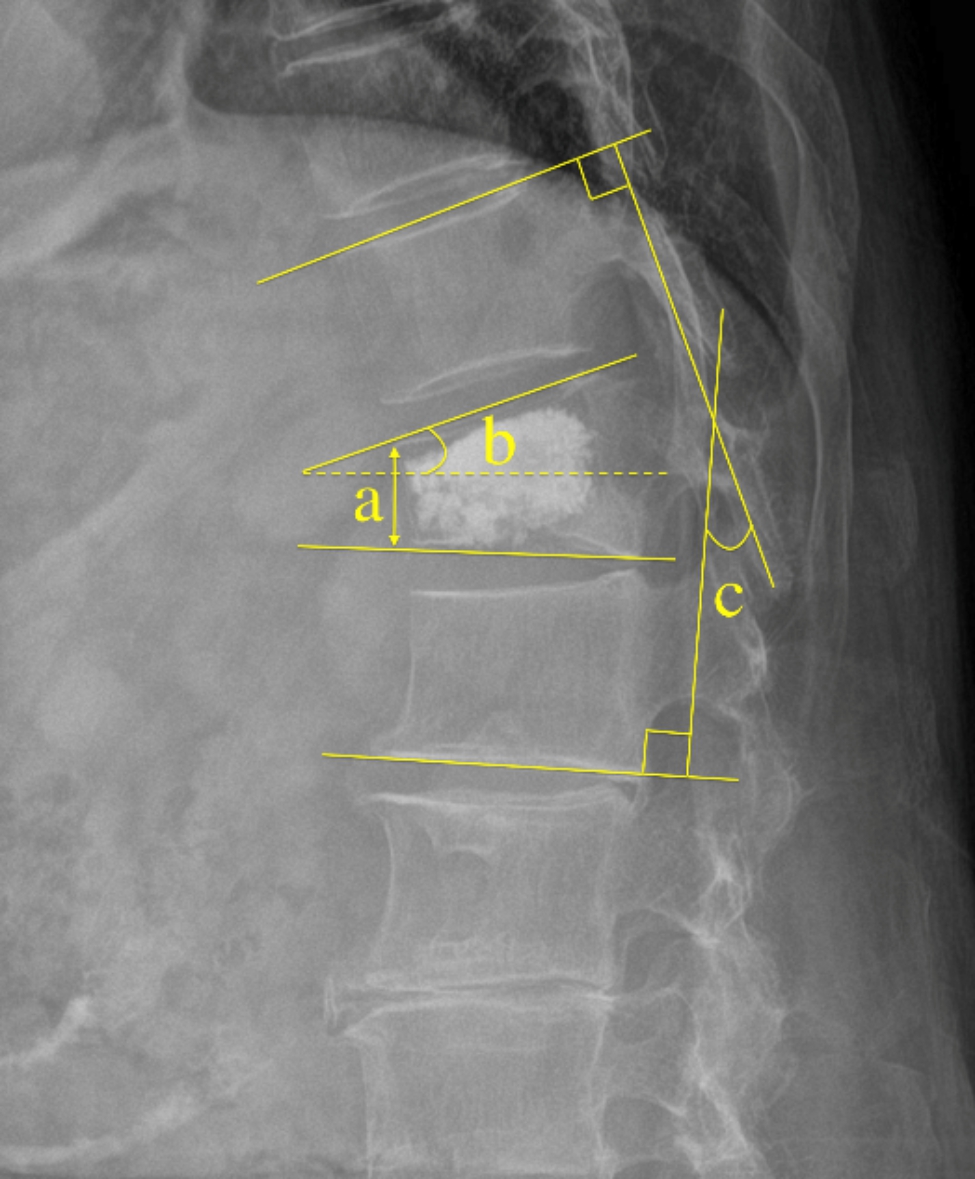
Measurement methods of related factors. **a**: AVH; **b**: kyphosis angle; **c**: Cobb angle

1. Systemic related factors: sex, age, body mass index (BMI), BMD, fracture segment, number of fractured vertebrae, history of diabetes, and history of hypertension.
2. Surgical related factors: surgical method (bilateral percutaneous kyphoplasty [PKP] or bilateral percutaneous vertebroplasty [PVP]), dosage of bone cement, shape of bone cement (“H” or “O” shape) [12], dispersion distribution grade of bone cement, leakage of bone cement, anterior vertebral height (AVH) restoration, kyphotic angle restoration, and Cobb angle restoration. All imaging data were X-ray revisited within 3 days after surgery.
3. To record the imaging measurements, all images were independently reviewed by two spine surgeons (YQ, XH; 3 and 8 years of clinical experience). In case of disagreement, consensus was achieved by group discussion with another surgeon (LZ;25 years of clinical experience). Definition and measurement of related factors: (1) shape of bone cement: group A (“H” shape), the filling pattern in the vertebral body involved two briquettes connected with / without a cement bridge and group B (“O” shape), the filling pattern in the vertebral body was a complete crumb and without any separation (Fig. 2); (2) dispersion distribution grade of bone cement: according to the X-ray after surgery. If the anteroposterior projection of bone cement was ≤ ½, and the lateral projection was ≤ ½, which was set as grade (1) If one of the two projections of bone cement was > ½, and the other was ≤ ½, which was set as grade (2) If both projections were > ½, which was set as grade 3 (Fig. 3). AVH restoration, kyphotic angle restoration, and Cobb angle restoration were calculated according to the preoperative and postoperative spinal lateral X-ray (Fig. 4).

### Statistical analysis

SPSS 22.0 version was used for statistical analysis of related risk factors. Measurement data are represented as median (interquartile range), namely M (P25 to P75), and categorical data are presented as percentages. Variables were tested for normality using the Shapiro–Wilk test. For data with normal distribution, differences between groups were analyzed using a t-test. The Mann–Whitney U test was used to compare data that were not normally distributed. A Chi-square test or Fisher exact test was used for enumeration data. Wilcoxon rank sum test was used for ranked data. *P* < 0.05 was considered statistically significant. R version 4.0.0 was used for nomogram model establishment and verification. Least absolute shrinkage and selection operator (LASSO regression) and logistic regression was used to screen the independent risk factors, and R was used to establish the risk prediction model of adjacent vertebral refracture after vertebroplasty. The area under the receiver operating characteristic (ROC) curve, calibration curve, and DCA were used to evaluate the prediction ability of the model.

## Results

### Comparison of systemic related factors

A total of 332 patients who underwent vertebroplasty in our hospital were included in the training cohort. After at least 2 years of follow-up, 43 patients had AVCF and the incidence of refracture was 13.0%. There were 289 non-refracture patients, including 227 females (78.5%) and 62 males (21.5%), and 43 refracture patients, including 36 females (83.7%) and seven males (16.3%). More women had primary and secondary vertebral fractures, but there was no significant difference in the ratio of male to female between the two groups (*P* > 0.05). The BMD of patients with refracture was significantly lower than that of patients without refracture (*P* < 0.001). There was no significant difference in age, BMI, history of hypertension and diabetes, fracture segment, or the number of fractured vertebrae between the two groups (*P* > 0.05), as shown in Table 1.

**Table 1:** Comparison of systemic factors between the non-refracture group and refracture group

	Non-refracture (n = 289)	Refracture (n = 43)	*P* value
Sex, n (%)			0.435
Female	227 (78.5%)	36 (83.7%)	
Male	62 (21.5%)	7 (16.3%)	
Age, (years)			0.224
< 70	113 (39.1%)	11 (25.6%)	
70–79	83 (28.7%)	17 (39.5%)	
≥ 80	93 (32.2%)	15 (34.9%)	
BMI, (kg/m^2^)			0.666
<18	23 (8.0%)	13 (30.2%)	
18–24	165 (57.1%)	9 (20.9%)	
24–28	85 (29.4%)	11 (25.6%)	
≥28	16 (5.5%)	10 (23.3%)	
BMD			<0.001
≤-3.9	31 (10.7%)	14 (32.5%)	
-3.9 – -2.5	103 (35.6%)	22 (51.2%)	
-2.5 – -1	130 (45.0%)	6 (14.0%)	
>-1	25 (8.7%)	1 (2.3%)	
Hypertension, n (%)			0.995
Yes	148 (51.2%)	22 (51.2%)	
No	141 (48.8%)	21 (48.8%)	
Diabetes, n (%)			0.726
Yes	54 (18.7%)	9 (20.9%)	
No	235 (81.3%)	34 (79.1%)	
Fracture segment, n (%)			0.058
T10	17 (5.9%)	6 (13.9%)	
T11–L2	187 (64.7%)	21 (48.8%)	
L3–L5	85 (29.4%)	16 (37.2%)	
Number of fractured vertebrae, n (%)			0.604
1	263 (91.0%)	38 (88.4%)	
2	22 (7.6%)	5 (11.6%)	
3	4 (1.4%)	0 (0%)	

### Comparison of surgical related factors

In the non-refracture group, 200 patients (69.2%) underwent PKP and 89 patients (30.8%) underwent PVP. In the refracture group, 29 patients (67.4%) underwent PKP, and 14 patients (32.6%) underwent PVP. There was no significant difference in surgical method between the two groups (*P* > 0.05). The proportion of leakage of bone cement in the refracture group (62.8%) was higher than that in the non-refracture group (17.6%) and the difference was statistically significant (*P* < 0.001). In terms of the distribution of bone cement in the vertebral body, the bone cement in the refracture group was more likely to show the “O” shape (69.8% vs. 31.5%; *P* < 0.001). However, there was no significant difference in dosage of bone cement, dispersion distribution grade of bone cement, AVH restoration, kyphotic angle restoration, or Cobb angle restoration (*P* > 0.05), as shown in Table 2.

**Table 2:** Comparison of surgery related factors between the non-refracture group and refracture group

	Non-refracture (n = 289)	Refracture (n = 43)	*P* value
Surgical method, n (%)			0.816
PKP	200 (69.2%)	29 (67.4%)	
PVP	89 (30.8%)	14 (32.6%)	
Dosage of bone cement, n (%)			0.084
< 4 ml	14 (4.8%)	1 (2.3%)	
4–6 ml	202 (69.9%)	26 (60.5%)	
>6 ml	73 (25.3%)	16 (37.2%)	
Shape of bone cement, n (%)			< 0.001
“O” shape	91 (31.5%)	30 (69.8%)	
“H” shape	198 (68.5%)	13 (30.2%)	
Dispersion distribution grade of bone cement, n (%)			0.576
Grade 1	8 (2.8%)	1 (2.3%)	
Grade 2	35 (12.1%)	4 (9.3%)	
Grade 3	246 (85.1%)	38 (88.4%)	
Leakage of bone cement, n (%)			<0.001
Yes	51 (17.6%)	27 (62.8%)	
No	238 (82.4%)	16 (37.2%)	
AVH restoration, (cm)	0.21 (0.13–0.31)	0.20 (0.10–0.30)	0.536
Kyphotic angle restoration, (°)	5.90 (4.60–7.20)	5.90 (4.70–7.50)	0.557
Cobb angle restoration, (°)	4.50 (3.60–6.00)	4.70 (3.50–6.20)	0.655

### Screening of related risk factors

**Fig. 5:**
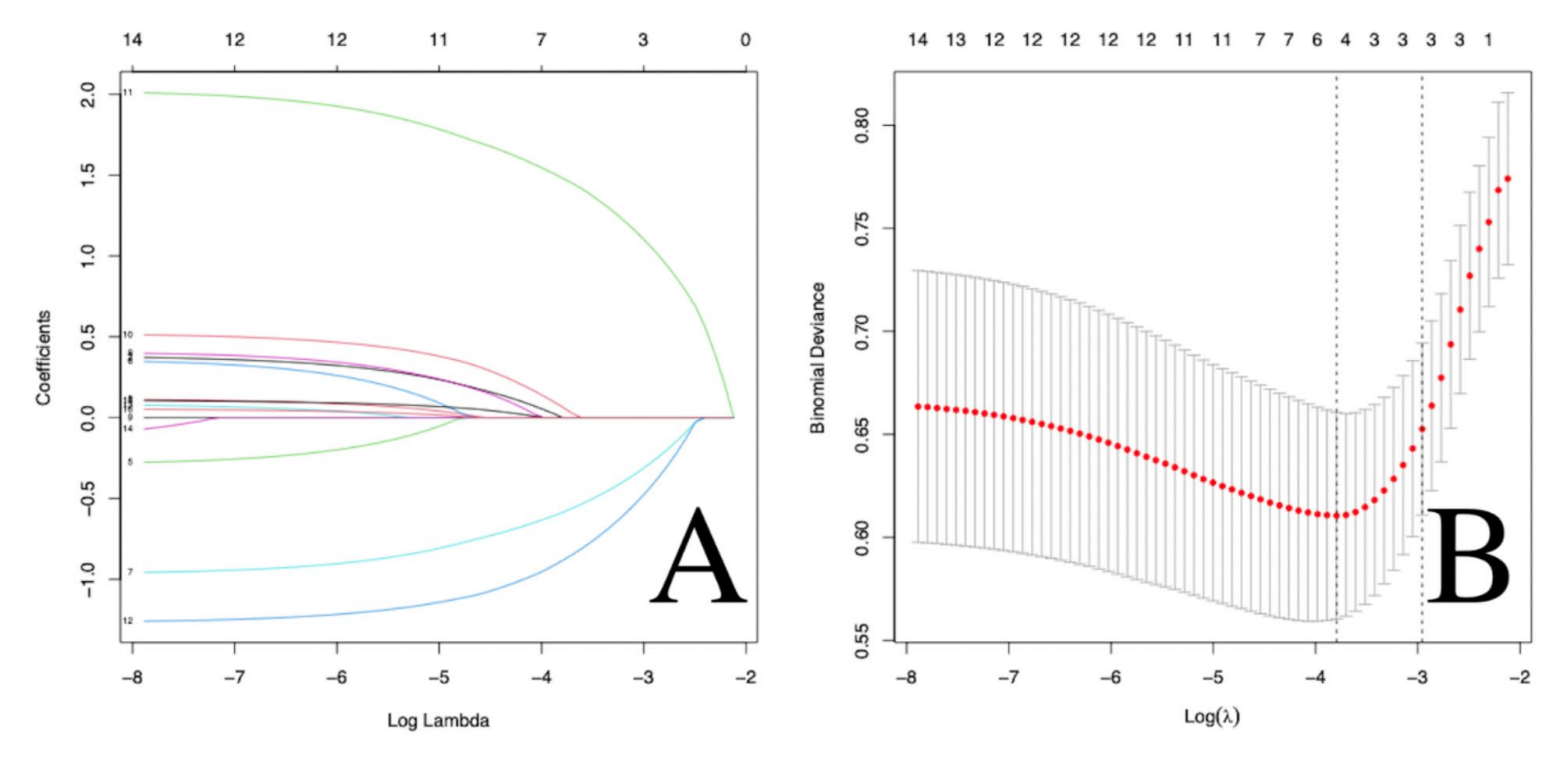
**(A)** LASSO coefficient profiles of the 16 features. **(B)** Optimal parameter (lambda) selection in the LASSO model using tenfold cross-validation via minimum criteria. Vertical line is drawn at the value selected using tenfold cross-validation, where the optimal lambda results in five features with nonzero coefficients. Curves in A correspond to risk factors: 11 = leakage of bone cement, 12 = shape of bone cement, 7 = BMD, 10 = dosage of bone cement, and 2 = age

LASSO regression analysis was performed using R 4.0.0 to screen relevant risk factors. Five factors related to age, BMD, bone cement dosage, bone cement leakage, and shape of bone cement were selected as risk factors for AVCF after vertebroplasty (Fig. 5). Multivariate logistic regression analysis was performed on the five selected related risk factors. Through logistic regression analysis, it was finally determined that low BMD, bone cement leakage and “O” shaped distribution of bone cement were independent risk factors for AVCF after vertebroplasty, as shown in Table 3.

**Table 3:** Results of multivariate logistic regression analysis of risk factors for refracture after vertebroplasty

	Regression	Standard	Wald		Odds	95% confidence interval	
Risk factor	coefficient	error	value	*P* value	ratio	Lower limit	Upper limit
Age			3.517	0.172			
70–79 vs. <70	-0.813	0.501	2.626	0.105	0.444	0.166	1.186
≥ 80 vs. <70	0.023	0.460	0.002	0.961	1.023	0.415	2.522
BMD			13.269	0.004			
≤-3.9 vs. >-1	2.012	1.134	3.150	0.076	7.476	0.811	68.958
-3.9– -2.5 vs. >-1	1.676	1.099	2.327	0.127	5.346	0.620	46.066
-2.5 – -1 vs. >-1	0.149	1.154	0.017	0.897	1.161	0.121	11.138
Leakage of bone cement (No)	1.951	0.393	24.626	< 0.001	7.033	3.255	15.197
Shape of bone cement (H)	1.419	0.407	12.133	< 0.001	4.132	1.860	9.180
Dosage of bone cement			1.713	0.425			
4–6 ml vs. <4 ml	-1.110	1.147	0.936	0.333	0.330	0.035	3.121
>6 ml vs. <4 ml	-0.463	0.424	1.190	0.275	0.630	0.274	1.446

### Establishment and verification of the nomogram clinical prediction model

**Fig. 6:**
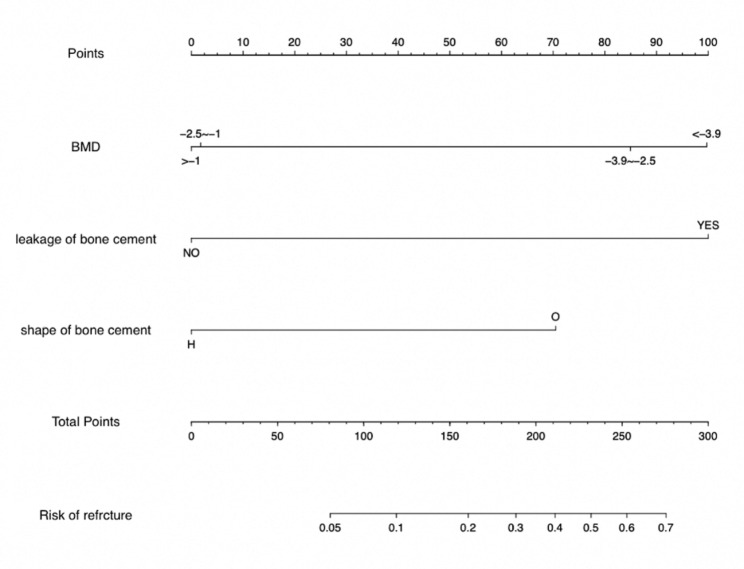
Nomogram prediction model for refracture after vertebroplasty

**Fig. 7:**
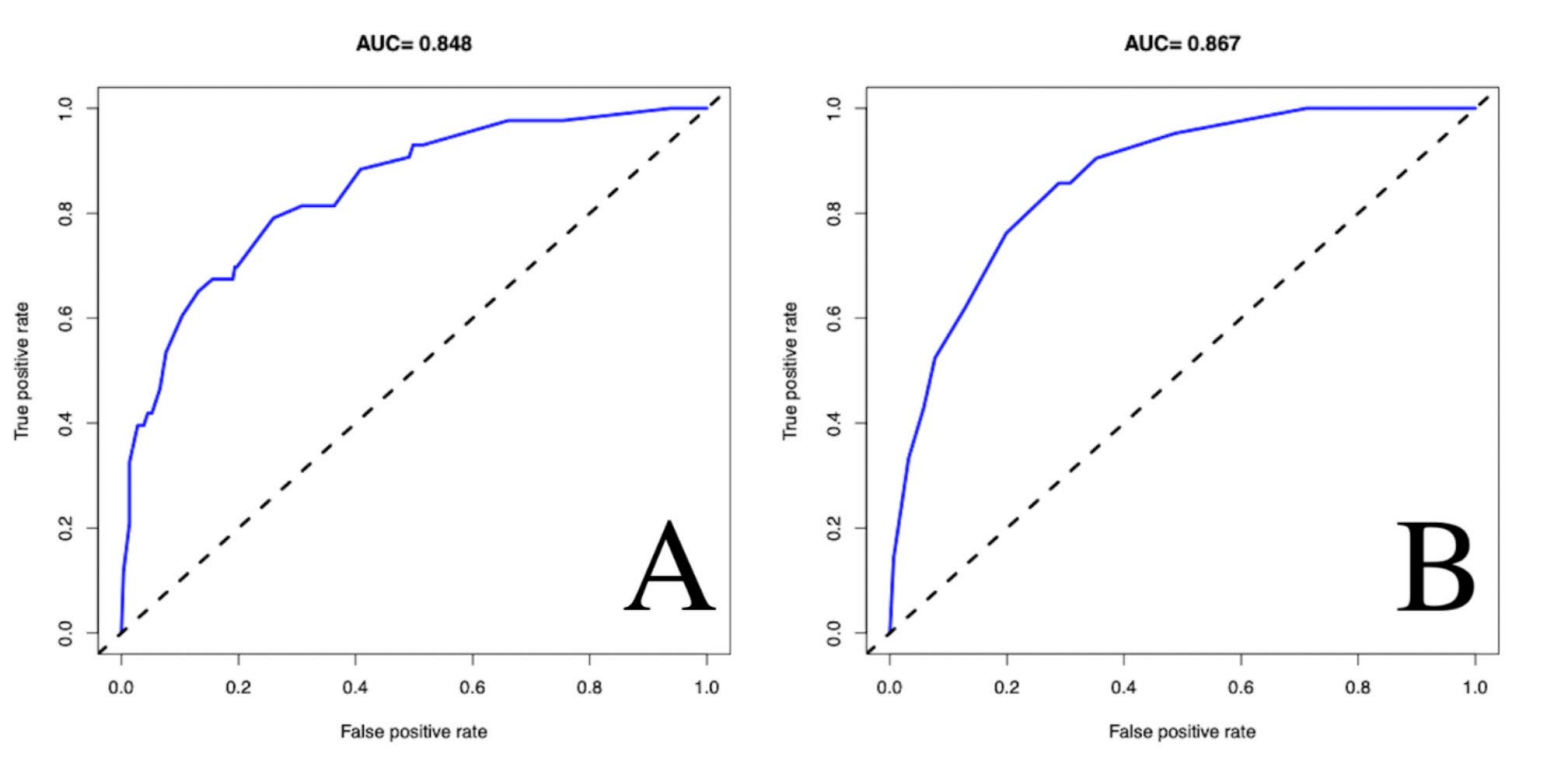
ROC curve and AUC of training cohort model (**A**) = 0.848 and validation cohort model (**B**) = 0.867

**Fig. 8:**
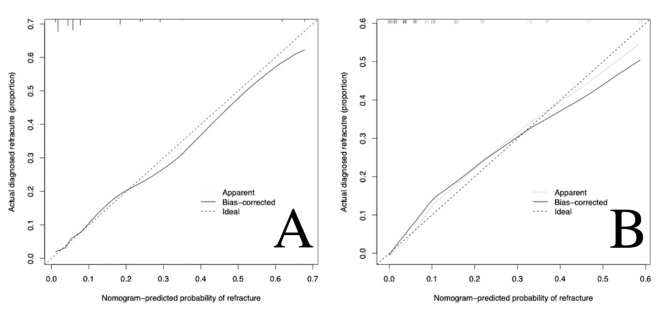
Calibration curves of the training cohort model (**A**) and validation cohort model (**B**), the X-axis represents the predicted risk of refracture and the Y-axis represents the actual diagnosed patients with refracture. The diagonal dotted line represents a perfect prediction by an ideal model. The solid line represents the performance of the nomogram, of which a closer fit to the diagonal dotted line represents a better prediction

**Fig. 9:**
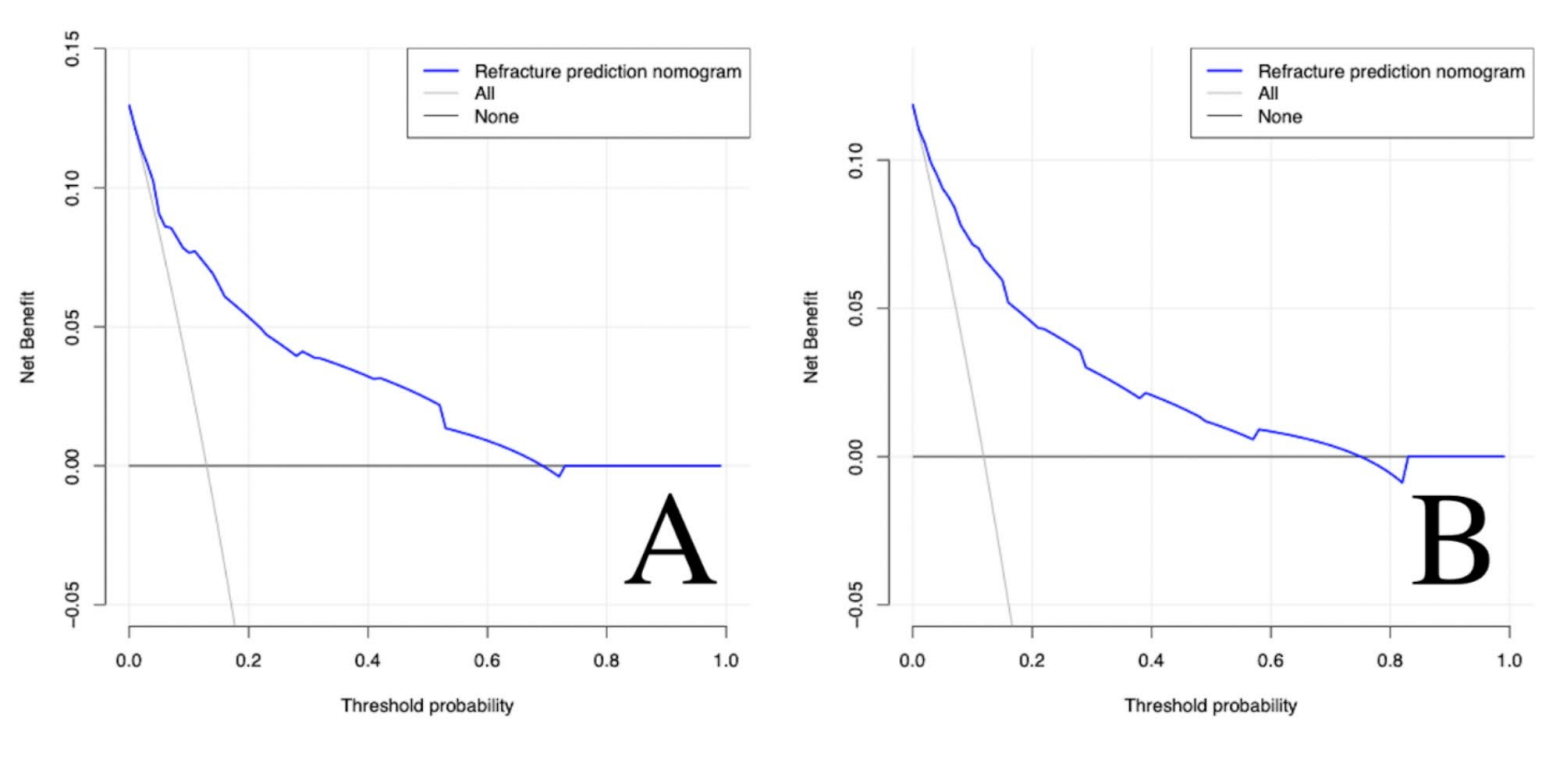
The x-axis measures the threshold probability. The y-axis measures the net benefit. The solid horizontal line represents the assumption that none of the patients have refracture. The oblique solid line represents the assumption that all patients have refracture. The blue solid line represents the refracture risk nomogram. The decision curve analysis of the training cohort model (**A**) shows that if the threshold probability of a patient and a doctor is 1–70%, using this prediction nomogram in the current study to predict refracture risk adds more benefit than the intervention-all-patients scheme or the intervention-none scheme. The threshold probability of validation cohort model (**B**) is 1–76%

According to the three independent risk factors of BMD, leakage of bone cement, and shape of bone cement, a nomogram clinical prediction model was established in the training cohort (Fig. 6). The area under the ROC curve (AUC) of the model was 0.848 (95%CI: 0.786–0.909) in the training cohort and 0.867 (95%CI: 0.796–0.939) in the validation cohort, showing good predictive ability of the model (Fig. 7). In both cohorts, the calibration curves showed a good agreement between the actual predictive ability and the optimal predictive level (Fig. 8). The DCA showed that the model has good clinical utilization within the whole threshold range (training cohort: 1–70%; validation cohort: 1–76%) (Fig. 9).

## Discussion

Vertebroplasty, as a minimally invasive treatment technique, is considered to be the first choice for the treatment of patients with OVCF, as it has the advantages of rapid pain relief and short recovery time [13–15]. However, AVCF is one of the major complications of vertebroplasty. There are many factors that may influence AVCF, such as sex, age, BMI, BMD, diffusion of bone cement, and dosage of cement [15, 16]. By multivariate logistic regression analysis, we found that low BMD, leakage of bone cement, and an “O” shaped distribution of bone cement were independent risk factors for AVCF after vertebroplasty.

Different distributions of bone cement during surgery have different effects on the stress on the vertebral body and adjacent vertebrae. Compared with the vertebral body with uniform distribution of bone cement, the vertebral body with inadequate distribution of bone cement showed significantly increased Von mises stress in vertical, flexion, extension, and lateral bending [17]. The excellent distribution of bone cement is an important factor influencing the clinical outcomes after vertebroplasty [18]. Patients will have better pain relief and fewer complications with higher dispersion distribution grades of bone cement [19]. Our study found that the bone cement in the refracture group was more likely to show the “O” shape (69.8% vs. 31.5%). A study by He et al. [12] showed that the distribution force mode of bone cement of the “H” shape was better than that of the “O” shape, so a “H” shaped distribution can achieve better clinical recovery in the short-term. Therefore, in order to reduce the refracture rate of adjacent vertebral bodies after vertebroplasty, surgeons should optimize the distribution of bone cement. According to our study, the puncture needle angle and the puncture depth should not be too large so as to avoid the formation of “O” shaped bone cement. If “O” shaped bone cement is found during the operation, an appropriate amount of puncture needle can be pulled out, part of the bone cement can be repaired, and the bone cement can be fully dispersed in the vertebral body as far as possible.

BMD, as measured by DXA, has been used for definition of osteoporosis since the mid-1990s [3]. Bone loss means a loss of vertebral strength, causing fragility fractures as a result of a slight fall or even sneezing [20]. There are many research studies that have shown that low BMD is a risk factor for AVCF after vertebroplasty [21–23]. In our study, the BMD of patients in the refracture group was significantly lower than that in the non-refracture group. All the patients in this study experienced low energy injury. Severe osteoporosis is the main cause of OVCF in most patients. The effects of degenerative bone changes on BMD are complex and many factors can lead to bone loss, such as advanced age, abnormal bone metabolism, and metabolic disease [24–26]. It is particularly important to carry out effective intervention for osteoporosis in patients after surgery. In a study of 192 patients with OVCF treated with antiosteoporosis drugs, the incidence of refracture was significantly lower in those who received regular therapy after 6 months of follow-up [27]. Therefore, anti-osteoporotic treatment should be a routine treatment in patients with OVCF who undergo vertebral augmentation, with the aim of decreasing the occurrence of AVCF.

Leakage of bone cement is one of the major complications of vertebroplasty [28, 29]. A large number of studies have shown that leakage of bone cement will increase the risk of AVCF after vertebroplasty [30–32]. Bone cement will aggravate the degenerative injury of the intervertebral disc which can change the stress distribution of the intervertebral disc, reducing its buffering effect [33]. Moreover, intradiscal cement leakage results in a more severe “pillarlike” effect on adjacent vertebra [34]. Consistent with previous studies, our study shows that the proportion of patients with leakage of bone cement in the refracture group (62.8%) was significantly higher than that in the non-refracture group (17.6%). A variety of risk factors for leakage of bone cement have been identified including intravertebral cleft, higher fracture severity grade, larger volume of bone cement, and low cement viscosity [35–38]. Prevention of bone cement leakage is an important initiative to reduce AVCF. It is recommended that the morphology, compression degree, and cortical integrity of the injured vertebrae are evaluated via imaging examinations before surgery to help determine the most reasonable surgical approach and depth, which is placement of the tip of the needle in the front one-third of the vertebral body or at least more than one-half, while keeping a safe distance from the vertebral wall and intravertebral cleft [39]. During the operation, the distribution of bone cement was dynamically monitored according to the fluoroscopy situation, and the injection speed was adjusted in a timely fashion. When bone cement leakage occurs, the injection should be stopped, and the bone cement volume and filling degree should not be excessively pursued.

In view of the high incidence of refracture in patients with clinical OVCF, it is particularly important to predict AVCF so as to carry out targeted prevention. In a previous study, Zhong et al. [32] established a fracture prediction scoring system according to the independent risk factors and Cox regression analysis to set the leakage of bone cement on a 4-point scale with preexisting fracture assigned 2 points. Results showed that a score of 0, 2, 4, and 6 corresponded to an incidence of subsequent fracture by 2 years of 3.3%, 8.7%, 19.9%, and 45.1%, respectively, and the c statistic of the validation model was 0.72. Our prediction model was based on BMD, leakage of bone cement, and shape of bone cement. All variables included in the nomogram were easy to determine. The model showed good predictive ability in that the AUC of the model was 0.848 and that of the validation model was 0.864. Our model has high prediction accuracy and selected predictors that can be considered as interventions during and after surgery. According to our study, in order to reduce the incidence of AVCF after vertebroplasty, we suggest that the bone cement should be sufficiently dispersed during the operation to avoid the stacking of bone cement. Leakage of cement can be avoided through detailed preoperative evaluation and careful intraoperative procedures. Long-term regular anti-osteoporosis treatment should be administered for patients with severe osteoporosis after surgery. It is of great significance to visually evaluate the risk of postoperative refracture and provide clinicians with a tool for predicting the occurrence of postoperative AVCF.

## Conclusion

We found that low BMD, leakage of bone cement, and an “O” shaped distribution of bone cement were independent risk factors for AVCF after vertebroplasty. The nomogram clinical prediction model established in this study has good prediction accuracy and clinical benefit.

## Data Availability

The datasets used and analyzed during the current study are available from the corresponding author on reasonable request.

## Abbreviations

OVCF: Osteoporotic vertebral compression fracture
AVCF: Adjacent vertebral compression fracture
LASSO: Least absolute shrinkage and selection operator
ROC: Receiver operating characteristic curve
DCA: Decision curve analysis
BMD: Bone mass density
AUC: Area under the curve
DXA: Dual energy X-ray absorptiometry
BMI: Body mass index
AVH: Anterior vertebral height
